# Bartonella henselae infection-mediated shunt nephritis 

**DOI:** 10.5414/CNCS111233

**Published:** 2024-01-04

**Authors:** Jennifer Nhan, Shriprasad Deshpande, Craig Futterman, Dong Hyang Kwon, Aadil Kakajiwala

**Affiliations:** 1 Pediatric Nephrology,; 2 Pediatric Critical Care,; 3 Pediatric Cardiology, Children’s National Hospital, and; 4 Pathology, MedStar Georgetown University Hospital, Washington, DC, USA

**Keywords:** membranoproliferative glomerulonephritis, endocarditis, shunt nephritis, ANCA vasculitis, full-house immunofluorescence

## Abstract

Serum anti-neutrophil cytoplasmic antibody (ANCA) positivity with membranoproliferative pattern on renal biopsy can be due to ANCA-associated vasculitis as well as chronic indolent infections. We present the case of an adolescent boy with congenital heart disease and history of cardiac surgery who presented with severe acute kidney injury requiring hemodialysis. Renal biopsy showed membranoproliferative glomerulonephritis with full-house immunofluorescence pattern. Low serum complements, PR3 ANCA positivity and elevated *Bartonella* immunoglobulin titers suggested a diagnosis of infective endocarditis-associated glomerulonephritis. Cardiac shunt revision and antibiotic therapy lead to improvement in kidney function. Chronic infections lead to formation of immune complexes that may cause deposit within the renal parenchyma and induce production of ANCA. The distinction of ANCA-associated vasculitis and chronic infections causing acute kidney injury is important in determining therapeutic management. While rare in the pediatric population, we highlight the importance in considering indolent infections in patients with acute glomerulonephritis and ANCA positivity, especially with risk factors.

## Case report 

### Presentation 

A 16-year-old boy presented with 2-weeks of fever followed by 1-week of lower extremity swelling, productive cough, and dyspnea. He developed generalized malaise and poor appetite with leg and facial swelling. Several days prior to presentation he had decreased urinary frequency with intermittent dark, tea-colored urine. There was no abdominal pain, vomiting, diarrhea, night sweats, or unexplained weight loss. On presentation, his initial vital signs were temperature of 36.7 °C, heart rate of 100 beats per minute, respiratory rate of 19 per minute, blood pressure of 120/90 mmHg, and oxygen saturation of 98%. The exam was significant for a surgical scar along the midline of his chest, 3/6 systolic murmur best heard over right sternal border with regular rate and rhythm, and edema of bilateral lower extremities up to the ankles. He was neurologically intact. No rashes or extremity clubbing were noted on exam. 

### Past medical history 

Medical history was significant for mirror image dextrocardia with double outlet right ventricle, ventricular septal defect (VSD), and pulmonic stenosis. At 1 year of age, he had a Rastelli procedure. The procedure included a VSD closure and a right ventricle to pulmonary artery conduit. At 6 years of age, he had subsequent conduit revision with 22 mm valved conduit. The patient’s most recent outpatient cardiology visit was 7 years prior, when he was noted to have conduit stenosis with peak velocity of 3.5 m/s and mean gradient of 25 mmHg. A 1.4 × 1.3 cm aneurysm was also noted at the proximal end of the conduit. 

### Investigation 

**Figure 1.**
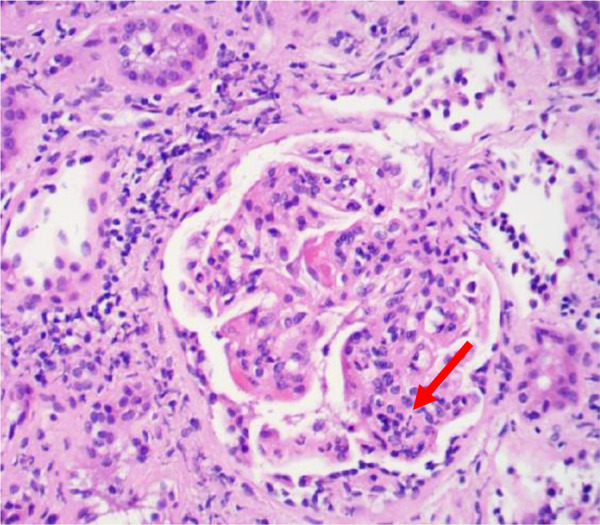
Light microscopy: proliferative glomerulus showing lobulation and neutrophilic infiltrate (arrow showing the neutrophilic infiltrate).

**Figure 2.**
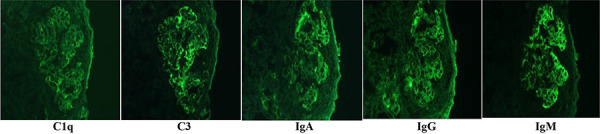
Immunofluorescence staining: positive for C1q, C3, IgA, IgG, and IgM antibodies, consistent with “full-house” pattern.

**Figure 3.**
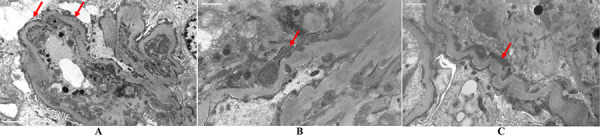
Electron microscopy. A: Fusion of podocytes with expansion of mesangial matrix. B: Subendothelial and rare subepithelial deposits with thickened basement membranes. C: Subendothelial deposits.

Blood testing initially showed a blood urea nitrogen level of 92 mg/dL and serum creatinine of 6 mg/dL with serum potassium of 6 mmol/L, bicarbonate of 12 mg/dL, calcium of 8 mg/dL, phosphorous of 8.3 mg/dL, and albumin of 1.8 g/dL. The C-reactive protein was normal at 0.65 mg/dL. Initial brain natriuretic peptide was elevated at > 5,000 pg/mL. Serum complement titers were C3 < 10 mg/dL and C4 < 7 mg/dL. Urine sample was not initially obtained due to anuria. He had a hemoglobin of 6 mg/dL and platelet count of 76,000/µL with no schistocytes and normal serum lactate dehydrogenase. Intact parathyroid hormone level was 59 pg/mL. Urine as well as anaerobic and aerobic blood cultures were negative. Anti-streptolysin O titers, anti-nuclear antibodies, and double-stranded DNA antibodies were negative. Given rapid decline in renal function, serum anti-neutrophil cytoplasmic antibody (ANCA) titers were checked. ANCA was positive with antiproteinase-3 (PR3) antibody titer of 21.8 AI (normal < 1.0 AI). A renal biopsy was performed (details below and in Figures 1, 2, and 3). Other infectious labs for active hepatitis B, C, human immunodeficiency virus, cytomegalovirus, and adenovirus were negative. It was noted that the patient had three cats (both indoor and outdoor). He had no recollection of cat bites or scratches. This history and concern for IE-medicated acute glomerulonephritis led to the testing of *Bartonella* antibodies and polymerase chain reaction (PCR). *Bartonella henselae* and *quintana* PCR were positive with *Bartonella henselae* immunoglobulin G (IgG) titers > 1:1,024. A summary of results is shown in Table 1. 

Initial echocardiogram showed moderate to severely decreased biventricular systolic function with left ventricular ejection fraction of 10%. A transthoracic echocardiogram showed additional severe tricuspid regurgitation, severe stenosis, and flow acceleration of the right ventricle to pulmonary artery conduit. There was a small residual ventricular septal defect with bi-directional shunting. There was no obvious large vegetation in proximal conduit; however proximal conduit, and valve leaflets were not well visualized. On transesophageal echocardiogram, an echodensity was noted along half the circumference of proximal conduit. There were no prior studies to discern acute versus chronic changes in the conduit. Cardiac catheterization showed a 55-mmHg gradient to the distal conduit and branch pulmonary arteries. A non-contrast cardiac magnetic resonance imaging was obtained to evaluate for subacute bacterial endocarditis or clot given conduit narrowing. There was a density in the supravalvular region between the pulmonary valve and stenosis that was undiscernible between calcification, thrombus, or vegetation. 

Abdominal ultrasound showed normal sized kidneys with increased echogenicity bilaterally and multiple left renal cortical cysts (largest measuring 7 mm). There was hepatosplenomegaly (liver on the left side measuring 16.8 cm and spleen on the right side measuring 19.7 cm). 

A computerized tomography of chest demonstrated non-specific focal subpleural opacity with peripheral ground glass changes. Bilateral pleural effusions were demonstrated along with compressive atelectasis. A bronchoalveolar lavage was performed which showed mixed cellular population with predominant macrophages and rare hemosiderin-laden macrophages without fungal organisms. 

### Renal biopsy 

Light microscopy: hypercellularity within the glomeruli with mesangial and endocapillary proliferation (Figure 1). There were no necrotizing, crescentic, or sclerosing lesions. There was mild tubulointerstitial disease.

Immunofluorescence: “full-house” positivity for IgG, IgA, IgM, C3, and C1q with 3+ granular capillary staining for IgM and C3 with co-localization of IgG, IgA, and C1q (2+ each) (Figure 2). Staining for ANCA antibodies was negative.

Electron microscopy: diffuse mesangial matrix expansion with increased cellularity and immune-complex deposits (Figure 3). Capillary basement membranes were thickened with frequent subendothelial and rare subepithelial deposits. There was significant podocyte fusion. 

### Differential diagnosis 

Membranoproliferative glomerulonephritis (MPGN) accounts for 7 – 10% of all biopsy-confirmed glomerulonephritis. [1]. MPGN is a pattern of glomerular injury with characteristic light microscopic changes of hypercellularity and thickening of the glomerular basement membrane [1]. There is characteristic mesangial hypercellularity, endocapillary proliferation, double-contour formation, duplication of basement membranes with immune-complex deposits in the subepithelial and subendothelial space [2]. 

MPGN is classified based on the immunofluorescence pattern on kidney biopsy [3, 4, 5] as either immune complex, complement-mediated, or neither. Immune-complex mediated (IC-MPGN) is characterized by subendothelial deposition of immunoglobulins and complement factors. IC-MPGN may occur when there are increased levels of circulating immune complexes and can be caused by autoimmune diseases, chronic infections, and paraproteinemia. Chronic infections leading to MPGN include hepatitis C and B, endocarditis, shunt infections, malaria, schistosomiasis, and mycoplasma [1]. 

Classically, shunt nephritis is described in patients who have an infected ventriculoperitoneal shunt but can occur with indolent chronic infections of other types of shunts or prosthesis [6]. Activations of the complement system can lead to hypocomplementemia. Although a rare cause of acute glomerulonephritis, shunt nephritis must be considered in cases with hypocomplementemia and “full-house” immunofluorescence pattern on renal biopsy. 

### Management 

Given the rapid decline in his renal function, he was initially treated with pulse steroids and needed intermittent hemodialysis for fluid overload. Ceftriaxone and vancomycin were initiated empirically for possible endocarditis but discontinued on hospital day 5 after multiple negative blood cultures. Positive PR3 antibody was concerning for ANCA vasculitis leading to therapeutic plasma exchange with replacement fluid mixture of albumin and fresh frozen plasma. 

With elevated *Bartonella henselae* titers and positive cat exposure, the diagnosis of *Bartonella*-induced shunt nephritis was established. He was treated with 2 weeks of rifampin and 6 weeks of doxycycline. He was treated with milrinone for concurrent heart failure. Medications were all renally dosed. After completion of the 2-week course of rifampin therapy, he underwent an elective conduit replacement which did not show any growth on culture, but on pathology showed fibro-adipose tissue with focal calcifications. Post surgery, he had complete recovery of his cardiac dysfunction with left ventricular ejection fraction of 61% and normal right ventricular function. While his peak creatinine was 6 mg/dL at the time of presentation, creatinine at time of discharge was 1.19 mg/dL. He had 1+ proteinuria and he remained off hemodialysis. By discharge, he was completing a prednisone wean and remained on 10 mg daily of amlodipine for hypertension. He was completing his course of doxycycline and was on sulfamethoxazole and trimethoprim for prophylaxis against *Pneumocystis jirovecii*. 

## Discussion 

Our patient with complex congenital heart defect presented with rapidly progressive glomerulonephritis associated with low complements and presence of PR3 antibodies. Renal biopsy showed a “full-house” immunofluorescence pattern with immune-dense deposits negative for PR3 antibodies. A more detailed history, the conflicting results on serology and renal biopsy, and history of cardiac conduit repair, suggested a diagnosis of shunt nephritis with positive IgG titers for *Bartonella*. Antibiotic therapy led to renal recovery and cessation of kidney replacement therapy. 

Shunt nephritis is an immune-complex complication of chronic infections related to cerebrospinal fluid and with infective endocarditis (IE) [7, 8]. Detection of ANCA is typically specific for ANCA-associated vasculitis (AAV). As reported by Ying et al. [8], various infections including endocarditis can infrequently induce production of ANCA [8]. In their retrospective study of 13 patients with PR3 ANCA-positive IE, nephropathy was seen in 4 patients. In various other cases, organisms reported to be isolated in IE-associated glomerulonephritis include *Staphylococcus aureus* (56% of cases), *Bartonella henselae*, *Propionibacterium acnes*, and *Coxiella burnetti* [6]. This shunt nephritis can clinically mimic other systemic disease such as AAV [9, 10]. As noted in our patient, the presence of low complements with positive ANCA is more suggestive of IE-associated glomerulonephritis rather than AAV [11]. Biopsy findings in IE-associated glomerulonephritis can demonstrate an immune-complex deposition or can be pauci-immune on immunofluorescence, making it hard to differentiate from AAV [12]. 

Cases of *Bartonella endocarditis* and acute glomerulonephritis have been reported in adults [11]. Diagnosis of *Bartonella* can be difficult and often presents as culture-negative IE. Diagnosis relies on serological testing of IgM and IgG antibodies. PCR has low sensitivity but is almost 100% specific for *Bartonella* infections. 

Treatment of IE-associated glomerulonephritis includes antimicrobial therapy to eradicate the infection [10, 13]. If there is ANCA positivity without clear clinical features to support AAV, it is prudent to consider chronic indolent infections that may cause immune-mediated glomerulonephritis via complement activation. 

## Conclusion 

ANCA positivity with MPGN can be due to AAV; however, in the clinical context of low complements and “full-house” immunofluorescence, indolent infections such as *Bartonella henselae* inducing immune complexes should be considered. Treatment of AAV and shunt nephritis differ and affect overall outcome of the patient. While still rare in the pediatric population, we highlight the importance in considering indolent infections like IE in patients with acute glomerulonephritis and ANCA positivity, especially when risk factors like cardiac surgery and congenital cardiac disease are present. 

## Funding 

There is no funding to disclose. 

## Conflict of interest 

Authors have no conflict of interest to disclose. 

**Table 1.** Summary of infectious disease and immunology/serology work-up.

**Infectious disease work-up**	
**Test**	**Result**
Initial blood aerobic and anaerobic cultures	No growth
Repeat blood aerobic and anaerobic cultures (3 days later)	No growth
4^th^ generation HIV test	Non-reactive
PPD skin test	No induration
QuantiFERON gold	Negative for tuberculosis
Infectious hepatitis panel	Negative
Hepatitis IgM antibody	Non-reactive
Hepatitis C antibody	Negative
Histoplasma urine antigen	Negative
Fungal antibody panel	Negative
*Blastomyces*, *Coccidioides*, *Histoplasma* antibodies	Negative
Cryptococcal antigen	Negative
Cytomegalovirus quantitative PCR	Negative
Adenovirus PCR	Negative
*Coxiella brunetti* IgG antibody	Negative
*Bartonella henselae* IgG antibody	> 1 : 1,024
*Bartonella henselae* IgM antibody	Negative
*Bartonella quintana* IgG antibody	1 : 256
*Bartonella quintana* IgM antibody	Negative
Whole blood *Bartonella* PCR	*B. quintana*: not detected
	*B. henselae*: detected
Respiratory bacterial and fungal cultures	Negative
*Pneumocystis jirovecci*	Negative
*Legionella pneumonia* culture	No growth
**Immunological/Serological work-up**	
**Test**	**Result**
C-reactive protein (CRP)	0.65 mg/dL
Complement C3	< 10 mg/dL
Complement C4	< 7 mg/dL
Anti-streptolysin O (ASO) titers	< 50 IU/mL
Antinuclear antibody	Negative
Double stranded DNA antibody	< 1 IU/mL
ANCA PR3 antibody	21.8 AI
ANCA myeloperoxidase antibody	< 1 AI
Smith antibody	Negative
RNP antibody	Negative
SSA/SSB	Negative
Antiphospholipid antibodies	Negative
Direct antiglobulin test/Direct Coombs	Negative
Antiplatelet antibodies	Negative
